# Evaluation of Trichodysplasia Spinulosa-Associated Polyomavirus Capsid Protein as a New Carrier for Construction of Chimeric Virus-Like Particles Harboring Foreign Epitopes

**DOI:** 10.3390/v7082818

**Published:** 2015-07-29

**Authors:** Alma Gedvilaite, Indre Kucinskaite-Kodze, Rita Lasickiene, Albertas Timinskas, Ausra Vaitiekaite, Danguole Ziogiene, Aurelija Zvirbliene

**Affiliations:** Vilnius University Institute of Biotechnology, V.A. Graiciuno 8, Vilnius LT-02241, Lithuania; E-Mails: alma.gedvilaite@bti.vu.lt (A.G.); indre.kodze@bti.vu.lt (I.K.-K.); rita.lasickiene@bti.vu.lt (R.L.); albertas.timinskas@bti.vu.lt (A.T.); ausra.vaitiekaite@bti.vu.lt (A.V.); danguole.ziogiene@bti.vu.lt (D.Z.)

**Keywords:** Chimeric virus-like particles, polyomaviruses, TSPyV VP1 protein, immunogenicity

## Abstract

Recombinant virus-like particles (VLPs) represent a promising tool for protein engineering. Recently, trichodysplasia spinulosa-associated polyomavirus (TSPyV) viral protein 1 (VP1) was efficiently produced in yeast expression system and shown to self-assemble to VLPs. In the current study, TSPyV VP1 protein was exploited as a carrier for construction of chimeric VLPs harboring selected B and T cell-specific epitopes and evaluated in comparison to hamster polyomavirus VP1 protein. Chimeric VLPs with inserted either hepatitis B virus preS1 epitope DPAFR or a universal T cell-specific epitope AKFVAAWTLKAAA were produced in yeast *Saccharomyces cerevisiae*. Target epitopes were incorporated either at the HI or BC loop of the VP1 protein. The insertion sites were selected based on molecular models of TSPyV VP1 protein. The surface exposure of the insert positions was confirmed using a collection of monoclonal antibodies raised against the intact TSPyV VP1 protein. All generated chimeric proteins were capable to self-assemble to VLPs, which induced a strong immune response in mice. The chimeric VLPs also activated dendritic cells and T cells as demonstrated by analysis of cell surface markers and cytokine production profiles in spleen cell cultures. In conclusion, TSPyV VP1 protein represents a new potential carrier for construction of chimeric VLPs harboring target epitopes.

## 1. Introduction

Viral structural proteins produced in heterologous expression systems and self-assembled to virus-like particles (VLPs) represent promising tools for modern vaccinology and diagnostics. Since the beginning of the 1980s, over 100 recombinant VLPs that originated from microbial, plant, insect, and mammalian viruses, belonging to 35 different virus families, have been constructed and characterized. Recombinant VLPs are extensively used for various applications, from basic virus assembly and structure studies to the production of human and animal vaccines [1]. The repetitive display of multiple epitopes on the VLPs makes them highly immunogenic [2]. Therefore, VLPs originating from almost all classes of viruses have been evaluated as carriers for generation of chimeric VLPs presenting foreign epitopes or large protein segments. Previous studies demonstrated that insertions of foreign protein segments at certain sites of VLP carriers derived from papilloma-, polyoma-, hepadna-, parvo- and retroviruses, as well as bacteriophages, did not disturb VLP assembly [3]. The first reported VLP carrier with inserted foreign epitopes was hepatitis B virus (HBV) core antigen (HBcAg) [4]. The advantage of HBcAg among other VLP carriers is its high-level synthesis and efficient self-assembly in virtually all known homologous and heterologous expression systems, including bacteria [5,6]. The HBcAg-derived chimeric VLPs have been successfully used for presenting HBV preS1 epitope, specific epitopes of hepatitis C virus, dengue virus, *Plasmodium falciparum*, tumor epitopes [7,8,9,10,11]. Another promising VLP carrier is hamster polyomavirus (HaPyV)-derived major capsid protein viral protein 1 (VP1) that is capable to form stable VLPs when produced in yeast expression system. HaPyV VP1-derived VLPs generated in yeast have been demonstrated to tolerate inserts of different size and origin at certain VP1 positions [12,13,14,15,16]. Four potential insertion sites located within the surface-exposed HI, BC, EF and DE loops of HaPyV VP1 molecule have been identified that tolerated simultaneous insertion of short-sized inserts, such as the 5 amino acid (aa)-long HBV preS1 epitope [12]. However, only two positions located at the HI loop and BC loop have been proven suitable for large-sized inserts, such as 45 aa-long and 120 aa-long segments of hantavirus nucleocapsid [13,14], as well as 99 aa-long segment of hantavirus glycoprotein [16]. A strong antibody response against hantavirus nucleocapsid segment presented on either HBcAg-derived or HaPyV VP1-derived chimeric VLPs was reported after VLP administration without any additional adjuvant [13,17]. Moreover, HaPyV VP1-derived chimeric VLPs harboring the 9 aa-long tumor epitope MUC1 have been demonstrated to induce both a strong epitope-specific antibody response and a cytotoxic T cell response in immunized mice [14,15]. 

Major capsid proteins VP1 of hamster, murine and human polyomaviruses (JCPyV, BKPyV) have been generated previously in yeast expression system [18,19]. Very recently, VP1 proteins of 11 newly discovered human polyomaviruses have been efficiently generated in yeast *Saccharomyces cerevisiae* and shown to self-assemble to VLPs [20]. Purified recombinant VLPs of trichodysplasia spinulosa-associated polyomavirus (TSPyV) VP1 protein were similar in their solubility and stability to HaPyV VP1-derived VLPs [18,20]. Therefore, in the current study we have exploited the TSPyV VP1 protein as a new carrier for insertion of foreign epitopes and evaluated its properties in comparison to HaPyV VP1 protein. As model epitopes for construction of chimeric VLPs, B cell-specific HBV preS1 epitope and a universal T cell-specific epitope (Pan HLA-DR epitope, PADRE) have been used. 

The preS1 epitope DPAFR spanning 31–35 aa residues of HBV preS1 protein has been identified as a recognition site of a neutralizing monoclonal antibody (MAb) MA18/7 [21,22]. The preS1 sequence is responsible for HBV binding to hepatocytes [23,24,25] and induction of virus neutralizing antibodies [26]. Neutralizing anti-preS1 MAb MA18/7 prevents binding of virus particles to cell membranes [23,24,27]. Previously, the preS1 epitope has been shown to induce a strong antibody response when inserted into HaPyV VP1 protein [12]. 

The PADRE epitope AKFVAAWTLKAAA is a 13 aa-long universal helper T cell peptide that is non-natural but is designed to have a high affinity for multiple DR alleles in humans and mice [28]. PADRE is a potent inducer of human CD4+ T cell proliferation [29], providing help for CD8^+^ cytotoxic T cells [28]. The epitope can also bind murine I-Ab and I-Ad MHC class II molecules [28]. Recent studies have demonstrated that PADRE as a part of chimeric proteins is responsible for the maturation and activation of dendritic cells [30]. The broad T cell activation properties of PADRE make it highly attractive for insertion into VLPs when developing novel vaccines.

In the current study, the preS1 and PADRE epitopes were inserted into either TSVyP VP1 protein or HaPyV VP1 protein at different positions and the resulted chimeric proteins were evaluated in terms of their capability to self-assemble to VLPs and immunogenicity in mice.

## 2. Materials and Methods

### 2.1. Bioinformatics Analysis of TSPyV VP1 Protein

Search of templates for structure modeling of recombinant polyomavirus VP1 proteins/pentamers, as well as of optimal sequence alignments of query sequence to template, were carried out using a sensitive profile-profile comparison method HHPRED [31]. Obtained sequence alignments were manually reorganized into alignments of pentamers (when pentamer template was available). In the case of asymmetric pentamer template, the appropriate alignment corrections were introduced. Structural models were produced following the protocol of comparative modeling by the use of a locally installed MODELER [32]. The inputs for the MODELER were a sequence alignment and the required structural template downloaded from the PDB repository [33]. Ten models for each case were generated with the MODELER. The results were examined using CHIMERA [34] tools and typical representatives for each group of results were selected.

### 2.2. Construction of Expression Plasmids

Construction of plasmids and all DNA manipulations were performed according to standard procedures [35]. Recombinants were screened in *E. coli* K12 DH5a strain. Cloning and expression in yeast of the entire TSPyV VP1-encoding sequence (GenBank: KP293746) optimized for yeast was described recently [20]. The TSPyV VP1-encoding sequence subcloned into the Eco32I site of the plasmid pUC57 was mutated by PCR-mediated insertion of a BglII cloning site and GSSG- or GSG-encoding linkers into the predicted regions of VP1: #1 (corresponding to aa 77–80) and #4 (corresponding to aa 279–281). Phusion High-Fidelity DNA Polymerase and primers with the following sequences were used for the experiments: 

TSV-VP1-1Bg-D, 5′-tcagatctaggttcttctggtcaagataaaccaacttctggt-3′,

TSVP1-1Bg-R, 5′-ctagatctgaaccagaagaaccattagcaacagtaactttttcag-3′,

TSVP1-4Bg-D, 5′-ggagatctaggttctggtgatatgcaatatagaggtttc-3′,

TSVP1-4Bg-R, 5′- ctagatctccagaaccaaccaaaaaaccaacaatatc-3′.

The nucleotide sequences of the mutated VP1-encoding variants: TSVP1-1Bg and TSVP1-4Bg were verified by DNA sequencing. The mutated TSVP1-1Bg and TSVP1-4Bg were then cloned into the XbaI site of the yeast expression vector pFX7 [18]. HaPyV VP1 genes encoding the modified VP1 for insertion of target sequences into either position #1 (corresponding to aa 80–89) or position #4 (corresponding to aa 288–295) with GSSG linker were described previously [12,15].

The four mutated VP1 gene variants (TSVP1-1Bg, TSVP1-4Bg, HaVP1-1Bg, and HaVP1- L4-Bg) inserted into pFX7 plasmid were digested with BglII, and the preS1 epitope-encoding and PADRE epitope-encoding oligonucleotide duplexes were introduced into each construct. The oligonucleotide duplexes were synthesized by Metabion (Martinsried, Germany). The insertion sites of the resulting recombinant plasmids were verified by DNA sequencing.

### 2.3. Generation, Purification and Electron Microscopy Analysis of Chimeric VLPs

The chimeric VP1 proteins of TSPyV and HaPyV were generated in yeast *Saccharomyces cerevisiae* strain AH22-214p (a, *leu2 his4 Δpep4*). Purification of chimeric proteins was performed as described previously [20,36]. Briefly, yeast cells were cultured in glucose-containing medium for 24 h and subsequently in galactose-containing induction medium for 18 h, then collected by centrifugation and stored at −20 °C until purification or analysis by electrophoresis in 12% sodium dodecyl sulfate-polyacrylamide gels (SDS-PAGE). For the analysis of target protein expression by SDS-PAGE, harvested yeast cells (20–50 mg) were suspended in 20–50 μL of DB150 buffer (150 mM NaCl, 1 mM CaCl_2_, 0.25 M L-arginine, 0.001% Triton X-100, 10 mM Tris/HCl, pH 7.2), lysed by vortexing with glass beads, mixed with the SDS-PAGE sample buffer (Thermo Fisher Scientific Baltics, Vilnius, Lithuania), boiled for 5 min and then subjected to SDS-PAGE. For purification of chimeric proteins, yeast cells producing recombinant proteins were suspended in DB450 buffer (450 mM NaCl, 1 mM CaCl_2_, 0.25 M L-arginine, 0.001% Triton X-100, 10 mM Tris/HCl, pH 7.2) with 2 mM PMSF and EDTA-free Complete Protease Inhibitor Cocktail tablets (Roche Diagnostics, Mannheim, Germany) and then homogenized with glass beads using Bead-Beater GB26 (BioSpec Products, Inc., Bartlesville, OK, USA). The chimeric proteins were purified from the supernatant of yeast cell lysate by ultracentrifugation overnight at 4 °C in 20%–60% sucrose gradient (100,000 × *g*). Fractions containing chimeric proteins were diluted in DB150 buffer and subjected to further ultracentrifugation at 100,000 × *g* for 18 h on CsCl gradient (1.23–1.38 g/mL). Purified chimeric proteins were collected, pooled and after dilution in DB150 buffer precipitated by ultracentrifugation for 4 h at 100,000 × *g*. Pellets containing recombinant chimeric proteins were dissolved in phosphate-buffered saline (PBS, pH 7.2), dialyzed against PBS, aliquoted (25, 50 and 100 μg) and then lyophilized or stored in PBS with 50% glycerol (at concentrations 1–2 mg/mL) at −20 °C until use. Concentrations of purified VLPs were determined using Bradford reagent (Sigma-Aldrich, St Louis, MO, USA) and by the densitometric scanning of the Coomassie brilliant blue-stained protein bands in SDS-PAGE using the ImageScanner III (GE Healthcare, Little Chalfont, UK) device. Protein concentrations in each preparation of chimeric VLP were determined using the ImageQuantTL software based on the comparison with known quantities of the purified unmodified VP1 proteins and bovine serum albumin (BSA, Sigma-Aldrich) run in the same gel. The unmodified TSPyV VP1 and HaPyV VP1 proteins were prepared as described previously [18,20].

To verify the VLP assembly of VP1-derived chimeric proteins, samples of each purified chimeric protein were placed on 300-mesh carbon-coated palladium grid. VP1-derived VLPs adhering to the grid were negatively stained with 2% aqueous uranyl acetate solution and examined with a Morgagni 268 electron microscope (FEI Inc., Hillsboro, OR, USA).

### 2.4. Immunization of Mice

The immunogenicity of chimeric VLPs was investigated by immunization of 8-week old female BALB/c mice obtained from the breeding colony at the Center for Innovative Medicine, Dept. of Immunology (Vilnius, Lithuania). Each group of mice (n = 3 per group) was immunized 2 times with different chimeric VLPs with or without adjuvant at 21-day intervals. For a primary immunization, mice were injected subcutaneously with 50 μg/mouse of chimeric VLPs either dissolved in PBS or emulsified in complete Freund’s adjuvant (CFA, Sigma-Aldrich). On day 21, the same groups of mice were immunized with chimeric VLPs (50 μg/mouse) either in PBS, or in incomplete Freund’s adjuvant (IFA, Sigma-Aldrich), respectively. Blood serum specimens were collected from a tail vein on day 21 after the first immunization and 14 days after the 2nd and the 3rd immunizations. Spleen cells for VLP stimulation were collected on day 21 after the 2nd immunization.

The work with experimental mice was performed by FELASA-certified personnel in accordance with the Lithuanian and European legislation. The Center for Innovative Medicine (Vilnius, Lithuania) has permissions for breeding experimental mice (license No. LT 59-902, permission No. 184) and their use for generation of polyclonal and monoclonal antibodies (permission No. 209).

### 2.5. Generation of Monoclonal Antibodies against Intact TSPyV VP1-Derived VLPs

Hybridomas producing monoclonal antibodies (MAbs) against TSPyV VP1 VLPs were generated essentially as described by Kohler and Milstein (1975) [37]. BALB/c mice were immunized with non-modified recombinant TSPyV VP1 VLPs with an adjuvant as described above. On day 28 after the 2nd immunization, the mouse with the highest TSPyV VP1-specific antibody titer was boosted with 50 μg of TSPyV VP1 VLPs in PBS. Four days later, spleen cells of the boosted mouse were fused with mouse myeloma Sp2/0 cells using PEG 1500 as a fusion agent (PEG/DMSO solution, HybriMax, Sigma-Aldrich). Hybrid cells were selected in growth medium supplemented with hypoxantine, aminopterin and thymidine (50× HAT media supplement, Sigma-Aldrich). Viable clones were screened by an indirect ELISA using TSPyV VP1 protein immobilized on the microtiter plates. Hybridoma cells were cultivated in complete Dulbecco’s modified Eagle’s medium (DMEM) containing 15% fetal bovine serum (FBS, Merck-Millipore, Darmstadt, Germany) and antibiotics. MAb isotypes were determined by ELISA using Mouse Immunoglobulin Isotyping Kit (BD Pharmingen, Franklin Lakes, NJ, USA). MAb specificity was tested by an indirect ELISA and Western blotting, as described below.

### 2.6. Indirect Enzyme-Linked Immunosorbent Assay (ELISA)

The levels of specific antibodies in blood sera of immunized mice were determined by an indirect ELISA using microtiter plates (Nunc MaxiSorp, Thermo Fisher Scientific, Waltham, MA, USA) coated with the respective antigens: chimeric VLPs, non-modified VLPs, synthetic biotinylated preS1 peptide (Pepscan, Lelystad, The Netherlands) and synthetic biotinylated PADRE peptide (Pepscan). Chimeric VLPs and non-modified VLPs were immobilized by adding 100 μL of the antigen solution (2 μg/mL) in coating buffer (50 mM Na-carbonate, pH 9.5) and incubation overnight at +4–8 °C. The plates were blocked for 30 min at room temperature (RT) with 1% BSA in PBS. For peptide immobilization, the plates were incubated overnight with streptavidin (Amresco, Solon, OH, USA) added at concentration 5 μg/mL in coating buffer, then blocked as above and incubated with the respective biotinylated peptide added at concentration 5 μg/mL in PBS with 0.1% Tween-20 (PBST). After antigen immobilization and blocking, the plates were rinsed with PBST and incubated for 1 h at RT with mouse antiserum specimens. Individual sera from mice were serially diluted in two-fold steps starting at 1:100 in PBST. Following additional rinsing, the plates were incubated with horse-radish peroxidase (HRP)-labeled anti-mouse IgG (Bio-Rad Laboratories, Hercules, CA, USA; 1:2000 in PBST) for 1 h at RT. After washing, the enzymatic reaction was detected with 3,3′,5,5′-tetramethylbenzidine (TMB) substrate (Sigma-Aldrich) and stopped by adding 1 M H_2_SO_4_. The optical density (OD) was measured at 450 nm in a microtiter plate reader (MULTISCAN GO, Thermo Fisher Scientific).

Titers of antigen-specific IgG antibodies were determined as the reciprocal of the highest antiserum dilutions giving OD_450_ values greater than three times the background. The background was calculated as the mean OD_450_ value plus 3 standard deviations (SD) for OD_450_ values of a preimmune mouse serum that was diluted 1:100 and added to the antigen-coated wells in triplicates. The preimmune serum was included into each ELISA plate.

### 2.7. SDS-PAGE and Western Blot Analysis

Recombinant proteins were fractionated by SDS-PAGE in 12% mini-gels and transferred to polyvinyldifluoride (PVDF) membranes (Merck-Millipore) under semidry conditions. Membranes were blocked with 1% gelatine in PBS for 1 h at RT, rinsed with PBST and incubated either with monoclonal antibody 3D10 [14] for detection of HaPyV VP1-derived chimeric proteins, or in-house developed monoclonal antibody against TSPyV VP1 protein (clone 17A5) for detection of TSPyV VP1-derived chimeric proteins for 1 h at RT. After rinsing in PBST, the membranes were incubated with HRP-labeled anti-mouse IgG (Bio-Rad Laboratories, 1:2000) for 1 h at RT. After several rinsing steps, the enzymatic reaction was visualized using TMB-blotting ready-to-use substrate (Sigma-Aldrich). Prestained protein molecular weight (MW) markers were purchased from Thermo Fisher Scientific Baltics.

### 2.8. Preparation of Murine Spleen Cell Cultures and Stimulation with VLPs 

The suspension of spleen cells from immunized mice was prepared by mechanical disruption. Ammonium chloride lysing buffer was used for the lysis of red blood cells. The prepared cells were seeded into 96-well cell culture plates at a density of 1 × 10^6^ cells/mL and stimulated either with VLPs (HaVP1-PADRE-4, TSPyV VP1 and HaPyV VP1) at a concentration 5 μg/mL or polyclonal activators Concavalin A (ConA) (Sigma-Aldrich) and lipopolysaccharide (LPS) (Sigma-Aldrich) added at concentrations 3 μg/mL and 1 μg/mL, respectively, to the growth medium: RPMI-1640 containing 10% FBS (Merck-Millipore) and antibiotics. After 24 h and 48 h of incubation, culture supernatants were collected and stored at −80 °C until analysis. 

Interleukin 12 (IL-12), interleukin 2 (IL-2) and interferon gamma (IFN-γ) concentrations in mouse spleen cell cultures were determined by sandwich ELISA using Mouse IL-12/IL-23 total p40 ELISA Ready-Set-Go, Mouse IFN gamma ELISA Ready-Set-Go (eBioscience, San Diego, CA, USA) and Mouse IL-2 ELISA set (BD Biosciences, San Jose, CA, USA) according to the recommendations of the suppliers.

### 2.9. ELISPOT Analysis

The frequency of IFN-γ-producing cells in the spleen cell cultures of immunized mice was determined by an enzyme-linked immunospot (ELISPOT) assay. Briefly, 100 μL of spleen cell suspension from the immunized mice were suspended in RPMI-1640 medium containing 10% FBS (Merck-Millipore) and antibiotics. The cells were seeded into sterile 96-well plates for ELISPOT assays (MAIPSWU10, Merck-Millipore) pre-coated overnight with anti-IFN-γ capture antibody (ELISpot development Module Mouse IFN-γ, R&D, MN, USA) in 100 μL of growth medium containing either ConA (3 μg/mL) or VLPs (HaVP1-PADRE-4, TSPyV VP1 and HaPyV VP1) at a concentration 5 μg/mL. Spleen cell cultures were diluted in two-fold steps starting from a density 0.5 × 10^6^ cells/well in duplicate. After 24 h of incubation at 37 °C in 5% CO_2_, spleen cells were removed and the plates were incubated with detection antibody (ELISpot development Module Mouse IFN-γ, R&D) for 8 h at +4–8 °C, followed by 2 h incubation with streptavidin-AP (Blue Colour Module, R&D). The BCIP/NBT substrate (Blue Colour Module, R&D) was used to visualize the enzymatic reaction. The immunostained spots were counted with CTL-ImmunoSpot^®^ S6 FluoroSpot Line counter (C.T.L., Cleveland, OH, USA) and analyzed using Immuno Capture 6.4 software (C.T.L.). For each mouse and stimuli, the ELISPOT count (spot count, SC) was reported as the average number of spots of replicates. The results were expressed as the number of responding cells per one million of spleen cells.

### 2.10. Flow Cytometry Analysis

A three color staining flow cytometry analysis of the murine spleen T cells was carried on the 28th day after the 3rd immunization with VLPs and re-stimulation *in vitro* (see above Section 2.8. Preparation of murine spleen cell cultures and stimulation with VLPs). The spleen cells were stained with: PE-conjugated anti-mouse CD4 (clone, RM4-5), Alexa Fluor 647-conjugated anti-mouse CD69 (clone, H1.2F3) (Biolegend, San Diego, CA, USA), FITC-conjugated anti-mouse CD8α (clone, 5H10) (Invitrogen, Carlsbad, CA, USA). After fixation the fractions of CD4^+^, CD8^+^ and CD69^+^ cells were measured using CyFlow Space (SysmexPartec GmbH, Germany) flow cytometer and analyzed using FlowJo, Version 10 software (FlowJo, Ashland, OR, USA).

### 2.11. Statistical Analysis

Statistical analysis of experimental data was performed using OriginPro 8 software (OrignLab, Northampton, UK). The *t-*test was used to compare the titers of insert-specific IgG antibodies between the groups of immunized mice and to compare cytokine levels between the untreated and VLP-treated murine spleen cell cultures. The differences were accepted as statistically significant if *p* < 0.05.

## 3. Results 

### 3.1. Computer Modeling of TSPyV VP1 Protein and Selection of Insert Positions

The potential insertion sites for foreign sequences within the TSPyV VP1 protein were determined based on the known three-dimensional fold structures of other polyomavirus VP1 proteins and successful exploring of HaPyV VP1 insertion sites located at the BC loop (site #1, aa 80–89) and HI loop (site #4, aa 288–295) [12,13]. 

Structure modeling of recombinant polyomavirus VP1 proteins/pentamers was performed using a sensitive profile-profile comparison method HHPRED. It has been found that the best structural template for modeling both native and recombinant versions of TSPyV VP1 protein is PDB:1sva–SV40 capsid (HHPRED Prob = 100.0, 56% identical VP1, lowest alignment gaping level). Though VP1 hexamer has been shown to be an asymmetric unit of the capsid, we have used the pentamer (a compact part) as a template. For modeling both native and recombinant versions of HaPyV VP1 protein the best template was PDB:1sid–murine polyomavirus (MPyV) capsid (HHPRED Prob = 100, 66% identical VP1, lowest alignment gaping level).

**Figure 1:**
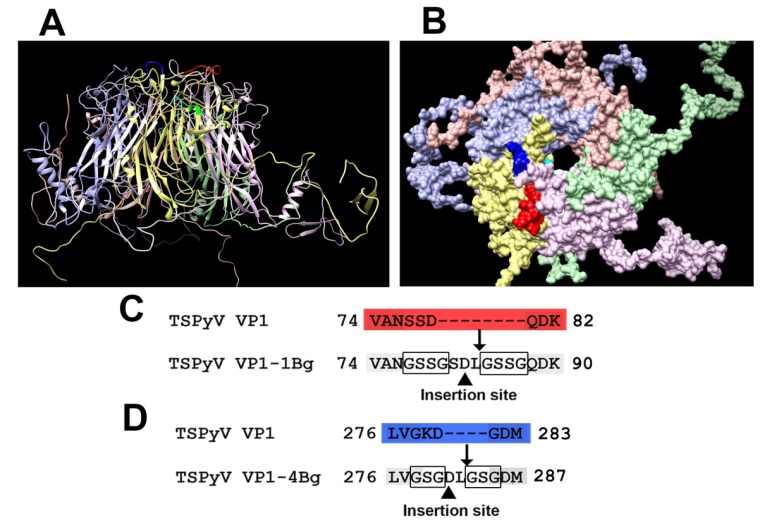
Molecular model of TSPyV VP1 pentamer and modification scheme of two surface-exposed loops: site #1 (aa 74–82) and site #4 (aa 276–283). The predicted structure of TSPyV VP1 pentamer is shown from the side (**A**) and the front (**B**); (**C**) modification scheme of site #1; (**D**) modification scheme of site #4. The site #1 is shown in red and the site #4 is shown in blue.

The generated three-dimensional molecular model of TSPyV VP1 pentamer with indicated surface-exposed sites #1 and #4 is presented in Figure 1A,B). The sites #1 and #4 are shown in red and blue, respectively. To insert target peptides, site #1 was modified by removing aa 77–79 of the TSPyV VP1 protein and inserting GSSG linkers surrounding the foreign peptide (Figure 1 C), while site #4 was modified by removing aa 279–280 and inserting GSG linkers surrounding the foreign peptide (Figure 1D).

**Figure 2:**
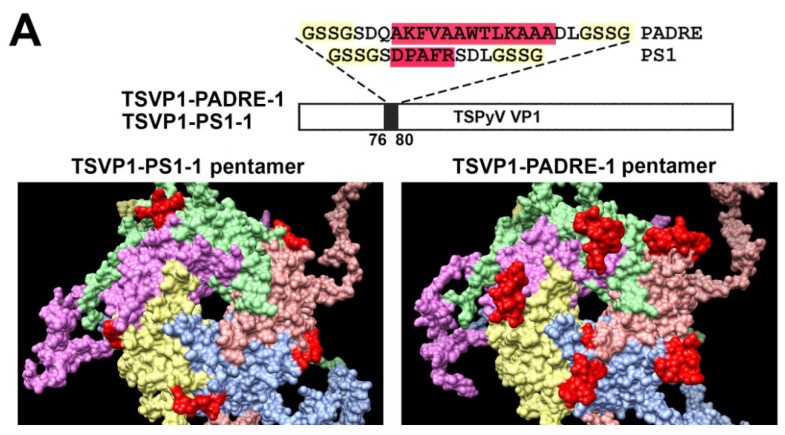

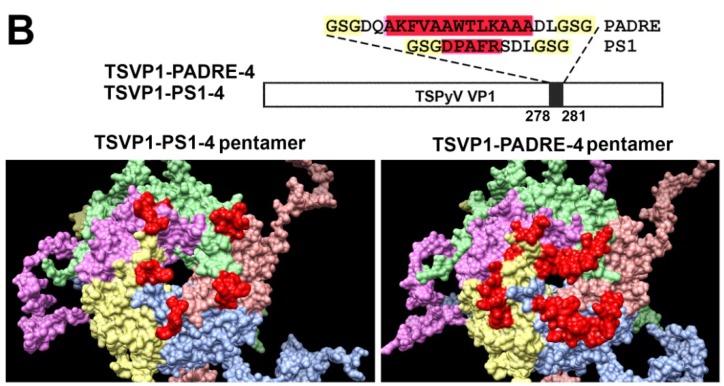
Molecular models of TSPyV VP1-derived chimeric proteins harboring either PS1 peptide (construct short name TSVP1-PS1) or PADRE peptide (construct short name TSVP1-PADRE) at site #1 ((**A**) constructs TSVP1-PS1-1 and TSVP1-PADRE-1) or site #4 ((**B**) constructs TSVP1-PS1-4 and TSVP1-PADRE-4). The inserts are shown in red.

**Figure 3:**
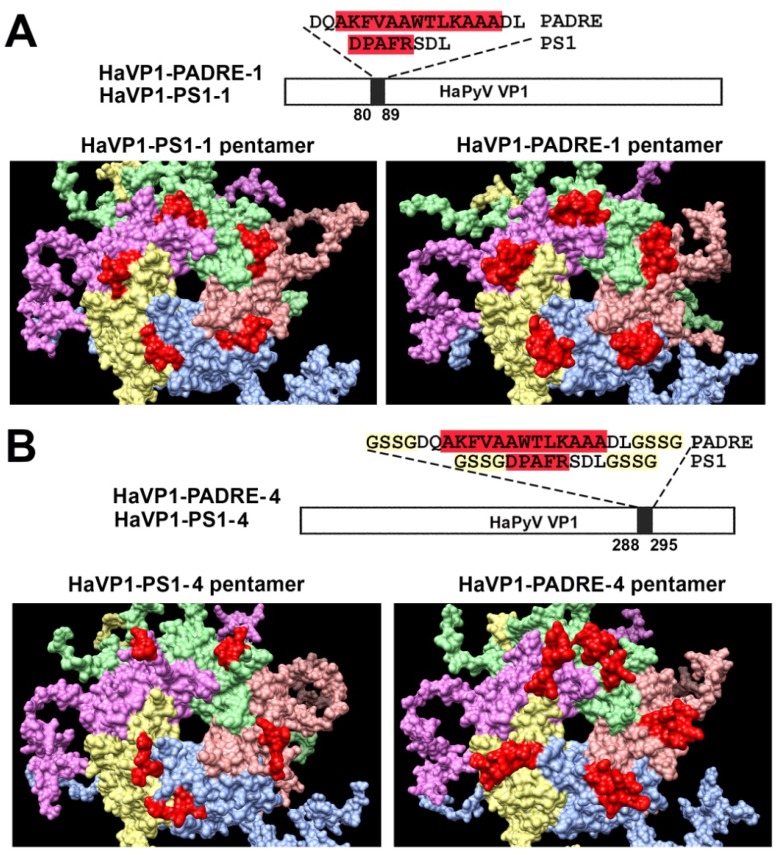
Molecular models of HaPyV VP1-derived chimeric proteins harboring either PS1 peptide (construct short name HaVP1-PS1) or PADRE peptide (construct short name HaVP1-PADRE) at site #1 ((**A**) constructs HaVP1-PS1-1 and HaVP1-PADRE-1) or site #4 ((**B**) constructs HaVP1-PS1-4 and HaVP1-PADRE-4). The inserts are shown in red.

Furthermore, molecular models of TSPyV VP1 pentamers with inserted 5 aa-long HBV PreS1 epitope (short name PS1) or a universal 13 aa-long T cell epitope PADRE either at site #1 (Figure 2A) or site #4 were generated (Figure 2B). In comparison, molecular models of HaPyV VP1 pentamers with the same inserts either at site #1 (Figure 3A) or site #4 were created (Figure 3B). Structural analysis of PADRE peptide using HHPRED method revealed that it potentially forms alpha-helical structure that might be a part of larger coiled-coil helix bundle. Due to this finding, additional structural template information has been added to alignments when modeling structure of recombinant VP1 pentamers with inserted PADRE epitope. The search for the best template for PADRE insert at site #1 of TSPyV VP1 protein while testing various versions of PADRE with N- and C-terminally flanking sequences revealed the best template to be the PDB: 1ug7 chain A (Prob = 85.4) and the best template for PADRE insert at site #4 of TSPyV VP1 protein–PDB:2mbh chain B (Prob = 41.7). In case of HaPyV VP1 protein, the best template for PADRE insert at site #1 of was selected PDB:4pnb chain A (Prob = 78) and the best template for PADRE insert at site #4 was selected PDB:1ug7 chain A (Prob = 59.5). The alignments of PADRE peptide with the mentioned templates were incorporated into the respective main alignments of recombinant VP1 to VP1 template. Ten models for each variant were generated and typical representatives were selected. As demonstrated in molecular models, the inserted peptides (marked in red) are localized on the surface of VP1 pentamers, both in case of TSPyV VP1 protein (Figure 2) and HaPyV VP1 protein (Figure 3). 

### 3.2. Construction and Purification of Chimeric VLPs

Based on the molecular model of TSPyV VP1 protein, potential surface-exposed positions for inserting peptides PS1 and PADRE were selected. The peptides were inserted into the TSPyV VP1 protein at either position #1 (aa 76–80) or position #4 (aa 278–281). The same peptides PS1 and PADRE were inserted into HaPyV VP1 protein at previously selected positions [12] - either position #1 (aa 80–89) or position #4 (aa 288–295). In total, eight different chimeric proteins were generated (Table S1). 

**Figure 4:**
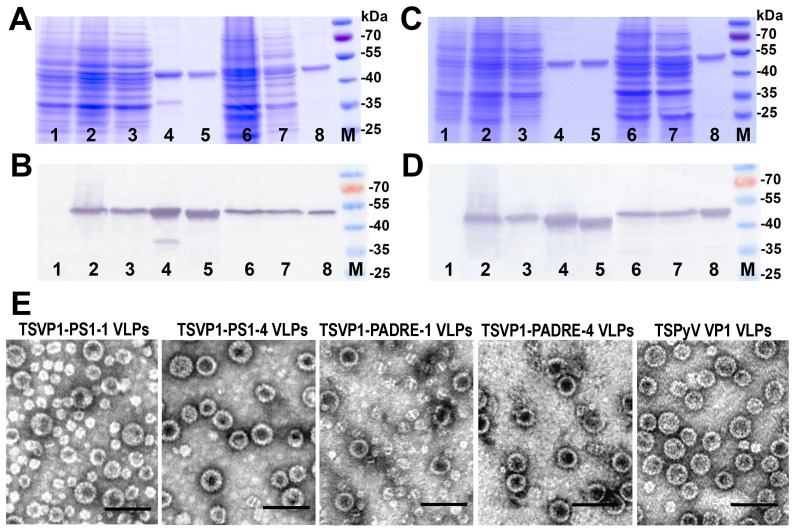
Analysis of the production of chimeric proteins TSVP1-PS1 (**A**,**B**) and TSVP1-PADRE (**C**,**D**) in yeast by SDS-PAGE (**A**,**C**) and Western blot with TSPyV VP1-specific MAb 17A5 (**B**,**D**). In all gels, the lysate of yeast cells transformed with an empty pFX7 plasmid (lane 1, negative control), the purified unmodified TSPyV VP1 protein (lane 5, positive control) and the pre-stained protein molecular weight marker (Thermo Fisher Scientific Baltics, lane M) were loaded. Chimeric TSVP1-PS1-1 ((**A**,**B**); lanes 2–4), TSVP1-PS1-4 ((**A**,**B**); lanes 6–8), TSVP1-PADRE-1 ((**C**,**D**); lines 2–4) and TSVP1-PADRE-4 ((**C**,**D**); lines 2–4) proteins were examined in the whole yeast cell lysates (lanes 2 and 6), lysate supernatants (lanes 3 and 7) and after purification (lanes 4 and 8). (**E**): Electron micrographs of TSVP1-PS1, TSVP1-PADRE and the unmodified TSPyV VP1 VLPs.

**Figure 5:**
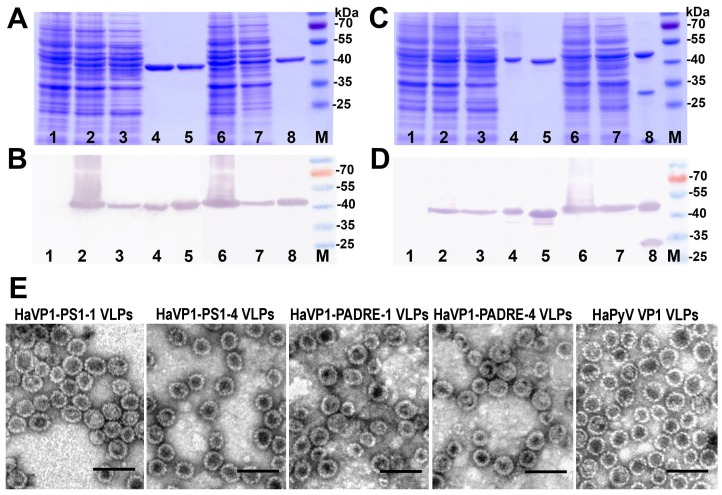
Analysis of the production of chimeric proteins HaVP1-PS1 (**A**,**B**) and HaVP1-PADRE (**C**,**D**) in yeast by SDS-PAGE (**A**,**C**) and Western blot with HaPyV VP1-specific MAb 3D10 (B,D). In all gels, the lysate of yeast cells transformed with an empty pFX7 plasmid (lane 1, negative control), the purified unmodified HaPyV VP1 protein (lane 5, positive control) and the pre-stained protein molecular weight marker (Thermo Fisher Scientific Baltics, lane M) were loaded. Chimeric HaVP1-PS1-1 ((**A**,**B**); lanes 2–4), HaVP1-PS1-4 ((**A**,**B**); lanes 6–8), HaVP1-PADRE-1 ((**C**,**D**); lines 2–4) and HaVP1-PADRE-4 ((**C**,**D**); lines 2–4) proteins were examined in the whole yeast cell lysates (lanes 2 and 6), lysate supernatants (lanes 3 and 7) and after purification (lanes 4 and 8). (**E**): Electron micrographs of HaVP1-PS1, HaVP1-PADRE and the unmodified HaPyV VP1 VLPs.

The expression of all recombinant constructs in yeast *S. cerevisiae* strain AH22-214p was analyzed by SDS-PAGE and Western blot using VP1-specific MAbs (Figure 4A,B). All chimeric proteins were found in the soluble fraction of yeast cell lysate, which allowed their purification using ultracentrifugation in sucrose and cesium chloride gradients (Figure 4A,B, lanes 3 and 7). The expression levels of TSVP1-PS1 and TSVP1-PADRE chimeric proteins were slightly lower than those of HaVP1-PS1 and HaVP1-PADRE chimeric proteins. The subsequent purification procedure of chimeric proteins revealed differences in their solubility depending on the inserted peptide. Yeast-expressed TSVP1-PS1 chimeric proteins were highly soluble as well as HaVP1-PS1 chimeric proteins. In contrast, TSVP1-PADRE chimeric proteins were less soluble than TSVP1-PS1 chimeric proteins and tended to form insoluble aggregates that precipitated at higher protein concentrations (>0.5 mg/mL). Similarly, HaVP1-PADRE chimeric proteins were less soluble than HaVP1-PS1 chimeric proteins although their aggregation was observed at higher concentrations as compared to TSVP1-PADRE chimeric proteins (>1.5 mg/mL). Furthermore, the purified HaVP1-PADRE-4 protein was not stable and showed a tendency to proteolytic degradation as detected by a fraction of two smaller peptides observed in SDS-PAGE and Western blot (Figure 5C,D, lane 8). The slightly lower expression level and solubility of the chimeric proteins with an inserted PADRE peptide affected the yield of the purified chimeric proteins considerably. The yield of TSVP1-PS1 chimeric proteins was comparable to that of the HaVP1-PS1 chimeric proteins: 0.62 and 0.95 mg from 1.0 g of wet yeast biomass, respectively. Moreover, the yield of TSVP1-PS1 chimeric proteins was only 1.6 times lower that the reported yield of the unmodified TSPyV VP1 protein (1.05 + 0.04 mg from 1.0 g of wet yeast biomass [20]. In contrast, the yield of TSVP1-PADRE chimeric proteins (0.14 mg from 1.0 g of wet yeast biomass) was significantly lower than the yield of HaVP1-PADRE chimeric proteins (0.56 mg from 1.0 g of wet yeast biomass) and even seven times lower than the yield of the unmodified TSPyV VP1 protein [20].

The influence of the insert position to the yield of the TSPyV VP1-derived chimeric proteins was not significant: an increase of protein yield by 0.07 mg from 1.0 g of wet yeast biomass was observed for the chimeric proteins harboring the PS1 insert at position #1 and similar increase was observed for chimeric proteins harboring the PADRE insert at position #4.

The ability of chimeric VP1-derived proteins to self-assemble into VLPs was examined using negative staining electron microscopy (EM). All eight chimeric proteins formed VLPs (Figure 4E and Figure 5E). HaVP1-PS1 chimeric VLPs were homogeneous and 45–50 nm in diameter (Figure 5E) that is typical for polyomavirus capsids and the unmodified recombinant HaPyV VP1-derived VLPs [12,18]. In contrast, the preparations of TSVP1-PS1-1 chimeric VLPs contained a mix of VLPs sized 45 and 20 nm in diameter while the preparations of TSVP1-PS1-4 VLPs contained a mix of VLPs sized 45 and 30 nm in diameter (Figure 4E). The inserted PADRE peptide even more influenced the size and assembly of chimeric VLPs. The EM analysis revealed not only 45 nm-sized VLPs but also smaller aggregates and pentamers in the preparation of TSVP1-PADRE chimeric protein (Figure 4E). HaPyV VP1-derived chimeric VLPs with inserted PADRE peptide were also heterogeneous in size, although chimeric VLPs HaVP1-PADRE-4 were less heterogeneous compared to HaVP1-PADRE-1 VLPs (Figure 5E).

### 3.3. Generation of Monoclonal Antibodies against TSPyV VP1 Protein and Their Use to Analyze the Structure of Chimeric VLPs

To analyze whether the selected insert positions are located on the surface of TSPyV VP1 protein, a collection of monoclonal antibodies (MAbs) against an intact (non-modified) TSPyV VP1 protein was generated. To generate hybridomas, spleen cells of the BALB/c mouse immunized with TSPyV VP1 VLPs were fused with myeloma cells. Hybrid clones were screened in ELISA for their reactivity with TSPyV VP1 VLPs. In total, 17 positive hybrid clones secreting TSPyV VP1-specific MAbs of IgG isotype were identified that were further stabilized by cloning and propagated in culture (Table S2). The reactivity of the MAbs (hybridoma supernatants) in Western blot was analyzed. Ten MAbs were reactive with TSPyV VP1 VLPs exclusively in ELISA, suggesting that they are directed to conformational epitopes. The MAbs were further tested in ELISA for their reactivities with chimeric TSPyV VP1 VLPs harboring inserted HBV preS1 and PADRE peptides either at position #1 or #4. Three out of 17 MAbs did not recognize chimeric VLPs, which indicates that the MAb epitopes are localized at insert positions (Table S2). One MAb (clone 2B4) did not recognize chimeric VLPs TSVP1-PADRE-1 and TSVP1-PS1-1 harboring peptide insert at site #1, and one MAb (clone 18A7 did not recognize chimeric VLPs TSVP1-PS1-4 and TSVP1-PADRE-4 harboring peptide insert at site #4. In addition, one MAb (clone 5E6) did not recognize any of chimeric VLPs indicating that its conformation-sensitive epitope is disturbed by any of the inserts. Localization of MAb epitopes at insert positions confirmed that the selected positions #1 and #4 are surface-exposed and accessible to B cells. 

### 3.4. Immunogenicity of Chimeric VLPs: Induction of Antibody Response

**Figure 6:**
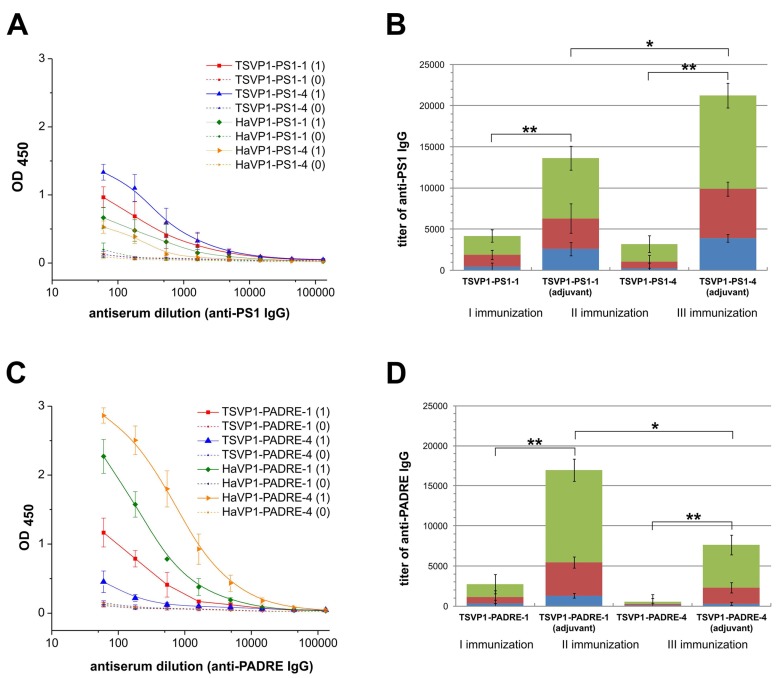
Antibody responses against the PS1 peptide (**A**,**B**) and PADRE peptide (**C**,**D**) in BALB/c mice (n = 3 per group) immunized with chimeric VLPs (TSVP1-PS1-1, TSVP1-PS1-4, TSVP1-PADRE-1, TSVP1-PADRE-4) as determined by an indirect ELISA. Left panel - titration curves of mouse serum before immunization (0) and on day 21 after the primary immunization (1) with adjuvant-emulsified chimeric VLPs harboring either the PS1 peptide (**A**) or PADRE peptide (**C**). Right panel–titers of PS1-specific (**B**) and PADRE-specific (**D**) IgG antibodies in groups of mice immunized with chimeric VLPs harboring either the PS1 peptide (**B**) or PADRE peptide (**D**) after the 1st, 2nd and the 3rd immunizations with or without an adjuvant. The asterisk (*) indicates statistically significant differences between the groups of immunized mice (*, *p* < 0.05; **, *p* < 0.01).

The immunogenicity of chimeric VLPs harboring either HBV preS1 (PS1) or PADRE epitopes at different positions was evaluated. For this purpose, the development of IgG antibodies against the inserted target peptides as well as against VP1 carrier was analyzed by an indirect ELISA in different groups of BALB/c mice immunized with chimeric VLPs. On day 21st after the primary immunization, IgG antibodies against the inserted peptides and the chimeric VLPs were detected in all groups of mice (Figure 6A,C, Table S3). High titers of VLP-specific antibodies (ranging from 900 to 108200) were determined in all groups of mice after the 1st immunization with an adjuvant (Table S3, rows 1–10). The groups of immunized mice (when chimeric VLPs were applied with an adjuvant) differed significantly in the levels of insert-specific IgG antibodies depending on the VLP carrier and the insert position (Figure 6, Table S3, rows 11–12). TSPyV VP1-derived VLPs with inserted PS1 epitope at position #4 (the HI loop) induced higher titers of epitope-specific antibodies (mean titer 3900) as compared to chimeric VLPs harboring the same PS1 peptide at position #1 (the BC loop, mean titer 2600) (Figure 6B, blue columns; Table S3, row 11). In contrast, PADRE peptide inserted at position #1 of the TSPyV VP1-derived VLPs was more immunogenic (mean titer 1300) as compared to PADRE peptide presented at position #4 of TSPyV VP1-derived VLPs (mean titer 300) (Figure 6D, blue columns; Table S3, row 12). The titration curves of mouse antisera after the primary immunization show that TSPyV VP1-derived chimeric VLPs induced higher levels of PS1-specific antibodies as compared to HaPyV VP1-derived VLPs (Figure 6A), while PADRE peptide was more immunogenic when presented on HaPyV VP1-derived VLPs, especially when inserted at position #4 (HaVP1-PADRE-4) (Figure 6C). To evaluate the secondary immune response indicative for immune memory, titers of insert-specific IgG antibodies after the 2nd and the 3rd immunizations either applied with the adjuvant or without any adjuvant were analyzed (Figure 6B,D). A significant increase of insert-specific IgG antibodies after repeated immunizations (when compared with the primary immunization) was observed in all groups of mice. After the 3rd immunization, IgG titers against the PS1 peptide increased by five-fold in mice immunized with TSVP1-PS1-1 and by 10-fold in mice immunized with TSVP1-PS1-4 chimeric VLPs without any adjuvant (Figure 6B, green columns). The titers of PADRE-specific IgG after the 3rd immunization increased by 50-fold and 2.5-fold, respectively, in mice immunized with TSVP1-PADRE-1 and TSVP1-PADRE-4 chimeric VLPs without an adjuvant (Figure 6D, green columns). As expected, significantly higher titers of peptide-specific IgG were determined in groups of mice immunized with chimeric VLPs emulsified in an adjuvant (Figure 6B,D). Similar increase of insert-specific IgG response after repeated immunizations of mice with HaPyV VP1-derived chimeric VLPs was reported previously [13,14,15]. 

Thus, the immunogenicity study showed that all chimeric VLPs were immunogenic in mice and induced activation of insert-specific B cells. 

### 3.5. Activation of Murine Spleen Cells by Chimeric VLPs

**Figure 7:**
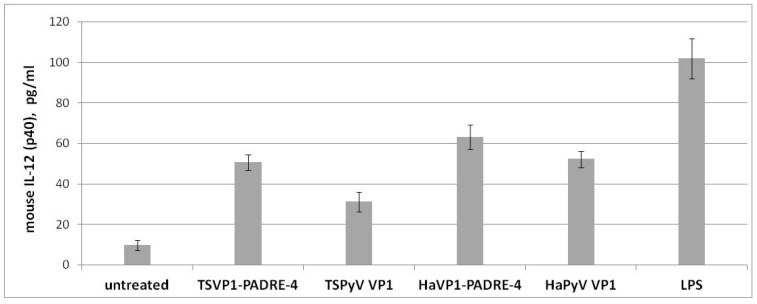
Production of IL-12 (p40) (pg/mL) by murine spleen cells incubated for 48 h with medium alone, TSVP1-PADRE-4 VLPs, unmodified TSPyV VP1 VLPs, HaVP1-PADRE-4 VLPs, unmodified HaPyV VP1 VLPs and LPS. Mean values and SD are indicated (n = 3).

To evaluate the ability of both chimeric and unmodified recombinant VLPs to activate antigen-presenting cells, the IL-12 (p40) secretion levels in spleen cell cultures of BALB/c mice were analyzed by ELISA. Both chimeric VLPs and intact VLPs added to murine spleen cultures induced high IL-12 secretion after 48 h of incubation (Figure 7) indicating activation of dendritic cells (DCs). The levels of IL-12 (p40) in VLP-treated spleen cell cultures were in the range of 31–63 pg/mL (Figure 7), which was comparable to that induced by *E. coli* lipopolysaccharide (LPS), a potent stimulator of dendritic cells used as a positive control (mean concentration 102 pg/mL). In contrast, the levels of IL-12 in spleen cell cultures incubated for 48 h without adding VLPs (negative control) were in the range of 7–12 pg/mL (Figure 7). Compared to the negative control, the IL-12 secretion level was increased two- to nine-fold by incubation with the VLPs, indicating the specificity of the VLP-mediated activation of spleen-derived dendritic cells. No significant differences between IL-12 levels induced by TSPyV VP1-derived and HaPyV VP1-derived VLPs were determined. 

Furthermore, the ability of spleen T cells derived from BALB/c mice immunized with chimeric VLPs harboring T cell-specific PADRE epitope to respond to restimulation with recombinant VLPs *in vitro* was analyzed. Activation of T cells was analyzed by ELISA, ELISPOT and flow cytometry assays. 

**Figure 8:**
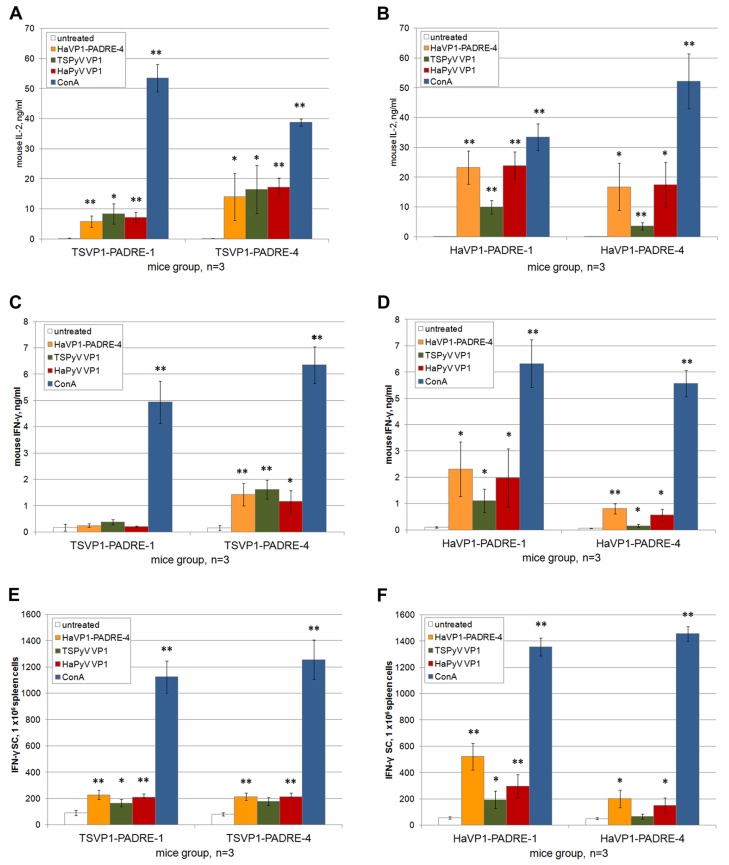
Analysis of T cell activation in spleen cell cultures of mice immunized with TSVP1-PADRE (left panel) and HaVP1-PADRE (right panel) VLPs. Production of cytokines IL-2 (ng/mL) (**A**,**B**) and IFN-γ (ng/mL) (**C**,**D**) by murine spleen cells incubated for 24 h with medium alone, HaVP1-PADRE-4 VLPs, unmodified TSPyV VP1 VLPs, unmodified HaPyV VP1VLPs and ConA was determined by ELISA (**A**–**D**). The number of IFN-γ-secreting cells treated in the same way was analysed by ELISPOT assay (**E**,**F**). Mean values and SD obtained from each group of immunized mice (indicated on the x axis of each picture) are given (n = 3). The asterisk (*) indicates statistically significant differences between the VLP- or ConA-treated and untreated spleen cell cultures (*, *p* < 0.05; **, *p* < 0.01).

The levels of T-cell specific cytokines IL-2 and IFN-γ were measured in spleen cell cultures treated either with chimeric VLPs or unmodified VLPs. Significant amounts of IL-2 (in the range of 3–23 pg/mL) and IFN-γ (in the range of 0.2–2.3 ng/mL) were determined in spleen cell cultures after 24 h of incubation with VLPs (Figure 8A–D). In contrast, the basal level of IL-2 and IFN-γ in spleen cell cultures incubated for 24 h without adding VLPs did not exceed 0.15 ng/mL and 0.17 ng/mL, respectively (Figure 8A–D). Thus, compared to spleen cell cultures incubated with medium alone, a 20.0- to 153.3-fold increase of IL-2 concentration and 1.1- to 13.5-fold increase of IFN-γ concentration following incubation with VLPs was observed. The highest production of IL-2 and IFN-γ was detected in groups of mice immunized with HaVP1-PADRE chimeric VLPs after stimulation with HaVP1-PADRE chimeric VLPs and the unmodified HaPyV VP1 VLPs. Antigen-specific stimulation of IFN-γ production was also observed in spleen cell cultures of mice immunized with TSVP1-PADRE chimeric VLPs after incubation with HaVP1-PADRE-4 and TSPyV VP1 VLPs. The ELISA results are in line with ELISPOT assay that revealed higher number of IFN-γ producing cells in murine spleen cell cultures in response to VLP stimulation (Figure 8E,F). A significant increase of IFN-γ spots (in the range of 90–500 spots/mln cells) was determined in spleen cell cultures after 24 h of incubation with VLPs (Figure 8E,F) while the basal level of IFN-γ-positive cells in spleen cell cultures incubated for 24 h without adding VLPs did not exceed 50 spots/mln cells (Figure 8E,F). Thus, compared to spleen cell cultures incubated with medium alone, a 1.8- to 10-fold increase of IFN-γ-positive cells after incubation with VLPs was observed.

In order to evaluate activation of memory T cells in immunized mice after *in vitro* stimulation with recombinant VLPs, three-color staining (CD4^+^ CD69^+^ and CD8^+^ CD69^+^) and subsequent flow cytometry analysis of spleen cell cultures was performed. The percentages of CD4^+^ CD69^+^ and CD8^+^ CD69^+^ cells were higher in VLP-treated spleen cell cultures as compared to untreated spleen cell cultures indicating activation of antigen-specific memory T cells (Table 1).

**Table 1:** The percentages (mean ± SD, n = 3) of CD4^+^ CD69^+^ and CD8^+^ CD69^+^ T cells in CD4^+^ and CD8^+^ populations of spleen cell cultures of BALB/c mice immunized with TSVP1-PADRE and HaVP1-PADRE chimeric VLPs.

**% of CD4^+^ CD69^+^ Cells within CD4^+^ T Cells Population**						
	**Antigen Used for Stimulation**	**Untreated**	**HaVP1-PADRE-4**	**TSPyV VP1**	**HaPyV VP1**	**ConA**
**Antigen Used for Immunization**						
**TSVP1-PADRE-1**		0.10 ±0.005	0.64 ± 0.05	0.58 ± 0.05	0.53 ± 0.09	24.42 ± 1.96
**TSVP1-PADRE-4**		0.11 ± 0.004	0.93 ± 0.1	0.57 ± 0.01	0.73 ± 0.05	21.64 ± 1.73
**HaVP1-PADRE-1**		0.10 ± 0.03	1.13 ± 0.15	0.65 ± 0.05	0.84 ± 0.09	18.4 ± 2.83
**HaVP1-PADRE-4**		0.12 ± 0.006	0.72 ±0.08	0.50 ±0.03	0.62 ±0.08	22.07 ±1.67
**% of CD8^+^ CD69^+^ Cells within CD8^+^ T Cells Population**						
	**Antigen Used for Stimulation**	**Untreated**	**HaVP1-PADRE-4**	**TSPyV VP1**	**HaPyV VP1**	**ConA**
**Antigen Used for Immunization**						
**TSVP1-PADRE-1**		0.67 ± 0.02	1.03 ± 0.09	0.77 ± 0.05	0.81 ± 0.09	32.44 ± 2.62
**TSVP1-PADRE-4**		0.64 ± 0.04	0.99 ± 0.07	0.86 ± 0.07	0.79 ± 0.15	33.10 ± 0.34
**HaVP1-PADRE-1**		0.65 ± 0.03	2.46 ± 0.41	1.03 ± 0.01	2.52 ± 0.25	27.70 ± 0.72
**HaVP1-PADRE-4**		0.68 ± 0.03	1.17 ± 0.11	0.74 ± 0.01	0.78 ± 0.09	26.11 ± 1.15

Murine spleen cells were incubated for 24 h with medium alone (negative control), HaVP1-PADRE-4 VLPs, the unmodified TSPyV VP1 VLPs, the unmodified HaPyV VP1 VLPs and ConA (positive control).

An increase of a percentage of CD69+ T cells (0.50%–1.13%) was determined within the CD4^+^ T cell population as compared to the untreated spleen cell cultures (0.1%–0.12%). Similar tendency was observed in CD8^+^ T cell population: higher percentage of CD69+ T cells (0.74%–2.52%) was determined within the CD8^+^ T cell population as compared to the untreated spleen cell cultures (0.64%–0.68%). However, the difference between the VLP-stimulated and the untreated CD8^+^ T cell population was less pronounced as compared to the CD4^+^ T cell population. More efficient activation of memory CD8^+^ T cells was observed in a group of mice immunized with HaVP1-PADRE-1 VLPs (2.46% and 2.52% of CD8+CD69+ cells after stimulation with HaVP1-PADRE-4 and HaPyV VP1, respectively), while only marginal increase of CD8+CD69+ T cells after VLP stimulation was determined in other groups of immunized mice (Table 1). 

Summarizing, the analysis of VLP-treated murine spleen cell cultures by different assays demonstrate that T cell-specific epitopes derived from recombinant VLPs are efficiently presented to T cells and induce their activation.

## 4. Discussion

Recombinant polyomavirus-derived VLPs that resemble native virions in size and shape are extensively used for targeted epitope presentation and show a great potential for vaccine development, gene delivery and diagnostics [1,38,39,40]. The first example of recombinant polyomavirus-derived VLPs was murine polyomavirus (MPyV) capsid protein VP1 generated by the high-yield expression in *E. coli* that allowed the subsequent *in vitro* assembly of VLPs under certain conditions [41,42]. Another well-established system for the production of VLPs is yeast *S. cerevisiae*. The first polyomavirus-derived VLPs produced in yeast were HaPyV VP1 VLPs [18]. Further studies have demonstrated the efficiency of yeast *S. cerevisiae*-based expression system for the production of VLPs originating from MPyV, SV40, BKPyV and JCPyV [19]. Recombinant polyomavirus-derived VLPs resemble viral virions that are around 45 nm in diameter and consist of 72 capsomers each containing five molecules of the major capsid protein VP1 [43]. The multimeric structure of polyomavirus-derived VLPs makes them an attractive platform for presenting target epitopes to the immune system. Moreover, recombinant VLPs have been shown to retain their morphological or immunological features after long-term storage and lyophilisation [13,42,44]. The repetitive structure of VLPs is crucial for inducing humoral immune response as demonstrated by vaccination of T cell-deficient mice with VLPs in comparison to capsomers [45]. Therefore, polyomavirus VP1-derived VLPs have been exploited to generate chimeric VLPs with surface-exposed target peptides of different length and origin to induce insert-specific antibody responses. In addition, the capability of chimeric VLPs to activate antigen-presenting cells and T cells has been demonstrated [1,38,39]. The enhanced immunogenicity of target peptides displayed on chimeric VLPs originating from rodent (MPyV, HaPyV) and monkey (beta-lymphotropic polyomavirus (LPyV) and SV40) polyomaviruses have been demonstrated [46,47]. Among other polyomavirus-derived chimeric VLPs, HaPyV VP1 protein is one of the most extensively investigated. The capability of HaPyV VP1-derived chimeric VLPs to tolerate long-sized inserts, such as 120 aa-long hantavirus nucleocapsid protein insert, as well as short-sized epitopes at 2 or even 4 insertion sites simultaneously have been demonstrated [12,13,14,15]. Thus, previous studies have proven the high insertion capacity of HaPyV VP1 protein for peptide epitopes. MPyV, LPyV and SV40 VP1-derived VLPs have been also exploited as carriers for foreign epitopes [18,48,49,50]. 

During the last decade, 11 novel human polyomaviruses have been identified [51,52,53,54]. Most recently, VP1 proteins of all 11 newly discovered human polyomaviruses have been efficiently produced in yeast *S. cerevisiae* in a form of VLPs [20]. Serological studies suggest that human polyomaviruses infect the general population with rates ranging from 35% to 90%. High seroprevalence of human polyomaviruses in clinically healthy persons suggests that they cause a subclinical latent infection while serious disease is only observed in patients with impaired immune functions [51]. No specific treatment or vaccines against polyomavirus-induced diseases are currently available. Recombinant chimeric VLPs with an enhanced immunogenicity designed on the basis of human polyomavirus-derived capsid proteins would be of special interest as vaccine candidates. However, VP1 proteins of the newly discovered human polyomaviruses have not yet been reported as target epitope carriers. 

One of the newly discovered human polyomaviruses is trichodysplasia spinulosa-associated polyomavirus (TSPyV) that causes a rare skin disease trichdysplasia spinulosa in immunocompromised patients [55]. TSPyV VP1 VLPs produced in yeast [20] have been shown to possess very high stability and solubility similar to those previously reported for HaPyV VP1 VLPs [18]. The exploitation of recombinant HaPyV VP1 protein as a carrier for different foreign epitopes indicated that the stability and solubility of the non-modified VP1 VLPs correlate with an efficient self-assembly and stability of VP1-derived chimeric VLPs as the inserted peptides may significantly affect VLP formation [12,13]. Among 11 yeast-produced VP1 VLPs of the newly discovered human polyomaviruses, TSPyV VP1 VLPs showed the highest stability and solubility suggesting that these VLPs might tolerate insertion of foreign peptides [20]. Therefore, in the current study TSPyV VP1 protein was chosen as a new carrier for peptide epitopes and was evaluated in regard to well-characterized epitope carrier HaPyV VP1 protein. Two types of peptides were selected for insertion into VLPs: the 5 aa-long HBV preS1 epitope DPAFR representing the B cell-specific epitope and the 13 aa-long PADRE epitope representing the T cell-specific epitope. The selected peptides differed significantly in their structure and hydrophobicity (Table S1). The potential insertion sites located on the surface of TSPyV VP1 pentamers were selected using computer modeling. As shown in the molecular model of TSPyV VP1 pentamer, the selected insert positions within the BC loop (aa 77–79) and HI loop (aa 279–280) allowed surface localization of the inserted peptides (Figure 2 and Figure 3). The surface exposure of the insert positions was also confirmed using a large collection of MAbs raised against the intact TSPyV VP1 protein: 3 out of 17 MAbs did not recognize chimeric VLPs harboring an insert either at site #1 or site #4 (Table S2). As B cells recognize surface-exposed epitopes, the non-reactivity of the MAbs in ELISA with the respective TSPyV VP1 protein–derived chimeric VLPs demonstrates that the conformation-sensitive MAb epitopes are disturbed by insertion. The EM analysis of chimeric proteins revealed differences in their self-assembly capacity depending on the insert: the VLPs with inserted PADRE peptide were more heterogeneous in size and shape that those with inserted HBV preS1 epitope. The observed differences might be explained by the content of hydrophilic and hydrophobic aa residues in the inserted peptide sequences (Table S1). Highly hydrophobic PADRE epitope (charge index 0) affected significantly VLP formation. As predicted form molecular models, PADRE peptide potentially forms alpha-helical structure that may cause VLP aggregation. In contrast, the hydrophilic HBV preS1 insert (charge index −1) was well tolerated both by TSPyV VP1 protein and HaPyV VP1 protein. These results are in line with previous studies where HBV preS1 peptide was well tolerated in chimeric VLPs originating from HaPyV VP1 and HBcAg even by simultaneous insertion at multiple sites [12,56]. Moreover, the EM analysis revealed that chimeric VLPs with PADRE peptide inserted at site #4 were more heterogeneous as compared to VLPs with PADRE insert at site #1 (Figure 4E and Figure 5E). Furthermore, the inserted hydrophobic PADRE peptide at position #4 of HaPyV VP1 protein affected its stability as demonstrated by SDS-PAGE and Western blot (Figure 5C,D, lane 8). Similar disruption pattern of HaPyV VP1-derived chimeric protein with an inserted CEA peptide and its flanking linker GSSG has been observed previously. The probable disruption site near position #4 has been mapped using VP1-specific antibodies [57].

In line with computer modeling and EM data, immunogenicity study revealed lower levels of PADRE-specific IgG antibodies in mice immunized with TSVP1-PADRE-4 chimeric VLPs as compared to TSVP1-PADRE-1 chimeric VLPs. PADRE peptide presented on HaPyV VP1-derived chimeric VLPs was more immunogenic, which may be explained by a more efficient VLP assembly. In addition, the immunogenicity of HaVP1-PADRE-4 correlates with lower structural flexibility of the inserts at site #4 (HI loop) of HaPyV VP1 modeled pentamer, thus suggesting that the flexibility of inserts plays a significant role in peptide presentation and activation of B cells. In contrast to chimeric VLPs with inserted PADRE epitope, the TSPyV VP1-derived chimeric VLPs with inserted 5 aa-long HBV preS1 epitope were more efficient in inducing insert-specific antibodies as compared to HaPyV VP1-derived chimeric VLPs. The immunogenicity data demonstrate the importance of the surrounding aa residues for the efficiency of peptide presentation to B cells.

All types of chimeric VLPs efficiently activated memory B cells as demonstrated in a long-term immunogenicity study that revealed a significant increase of insert-specific IgG titers after repeated injections, even when the VLPs were applied without any adjuvant. As an activation of T helper cells is necessary for inducing memory B cell response and immunoglobulin class switch, the long-term immunogenicity data indicate an efficient T helper activation by chimeric VLPs. Moreover, the ability of all chimeric VLPs to activate dendritic cells and T cells was demonstrated by enhanced expression of cytokines IL-12, IL-2 and IFN-γ, as well as T cell activation-related surface marker (CD69) in the cultures of murine spleen cells treated with chimeric VLPs. 

Summarizing, the TSPyV VP1-derived recombinant VLPs are immunogenic and represent an efficient carrier for foreign epitopes. To our knowledge, this is the first study that describes chimeric VLPs derived from the capsid protein of the newly discovered human polyomaviruses. 

## 5. Conclusions

TSPyV VP1 protein represents a new carrier for insertion of foreign epitopes at surface-exposed positions within the HI or BC loop. The yield and stability of chimeric TSPyV VP1-derived VLPs depends on the position and hydrophobicity of the insert.

A large collection of novel MAbs against TSPyV VP1 protein has been generated. The selective reactivity of the MAbs with TSPyV VP1-derived chimeric VLPs confirms the surface exposure of the inserts.

All chimeric VLPs are immunogenic and induce formation of insert-specific IgG antibodies in immunized mice. An increase of the titers of insert-specific IgG antibodies after repeated immunizations indicates immune memory, even when the VLPs are applied without any adjuvant.

All chimeric VLPs induce activation of dendritic cells and T cells in murine spleen cells cultures as demonstrated by the analysis of cytokine production profiles and T cell surface marker CD69.

The immunogenicity of TSPyV VP1-derived chimeric VLPs is similar to that observed with HaPyV VP1-derived chimeric VLPs. However, highly hydrophobic PADRE epitope more disturbs VLP assembly and induces lower antibody response when inserted into TSPyV VP1 protein as compared to HaPyV VP1 protein. In contrast, the HBV preS1 epitope is more immunogenic when presented on TSPyV VP1-derived chimeric VLPs.

The current study provides the first example of chimeric VLPs constructed on the basis of the capsid protein of the newly discovered human polyomaviruses.

## Supplementary Files

Supplementary File 1
