# Supplementary material for: Evaluation of Trichodysplasia Spinulosa-Associated Polyomavirus Capsid Protein as a New Carrier for Construction of Chimeric Virus-Like Particles Harboring Foreign Epitopes
Source: Viruses. 2015 Jul 29;7(8):4204–29. doi: 10.3390/v7082818 (PMC4576179; doi:10.3390/v7082818)
Supplement: Supplementary File 1 [file viruses-07-02818-s001.pdf]

Supplementary Information

Table S1. The list of recombinant chimeric proteins with inserted HBV preS1 (PS1) and PADRE peptides.

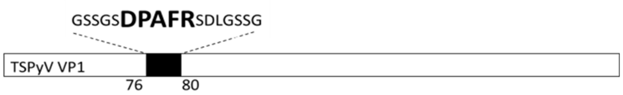

Construct	TSVP1-PS1-1
Insert position	#1
Insert	GSSGSDPAFRSDLGSSG
Target peptide	DPAFR
Charge	-1
Attribute	acidic

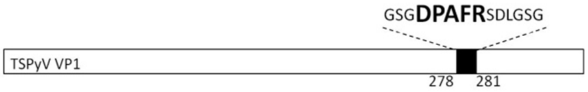

Construct	TSVP1-PS1-4
Insert position	#4
Insert	GSGDPAFRSDLGSG
Target peptide	DPAFR
Charge	-1
Attribute	acidic

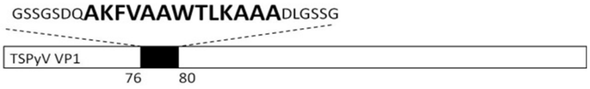

Construct	TSVP1-PADRE-1
Insert position	#1
Insert	GSSGSDQAKFVAAWTLKAAADLGSSG
Target peptide	AKFVAAWTLKAAA
Charge	0
Attribute	neutral

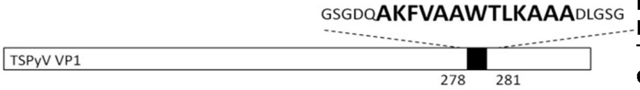

Construct	TSVP1-PADRE-4
Insert position	#4
Insert	GSGDQAKFVAAWTLKAAADLGSG
Target peptide	AKFVAAWTLKAAA
Charge	0
Attribute	neutral

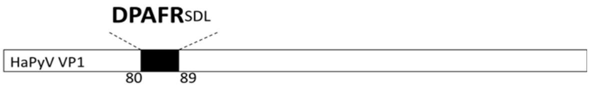

Construct	HaVP1-PS1-1
Insert position	#1
Insert	DPAFRSDL
Target peptide	DPAFR
Charge	-1
Attribute	acidic

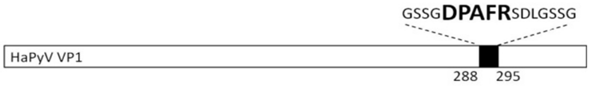

Construct	HaVP1-PS1-4
Insert position	#4
Insert	GSSGDPAFRSDLGSSG
Target peptide	DPAFR
Charge	-1
Attribute	acidic

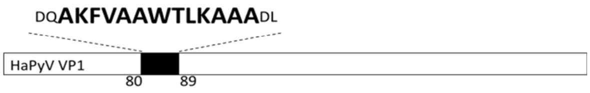

Construct	HaVP1-PADRE-1
Insert position	#1
Insert	DQAKFVAAWTLKAAADL
Target peptide	AKFVAAWTLKAAA
Charge	0
Attribute	neutral

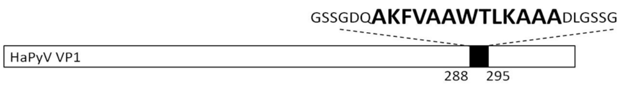

Construct	HaVP1-PADRE-4
Insert position	#4
Insert	GSSGDQAKFVAAWTLKAAADLGSSG
Target peptide	AKFVAAWTLKAAA
Charge	0
Attribute	neutral

* Charge index was determined using GenScript Peptide Property Calculator (GeneScript). Hydrophobic uncharged aa residues are indicated in green, basic aa residues in blue, acidic aa residues in red and other aa residues in black.

Table S2. Isotypes and antigen-binding activity of MAbs raised against TSPyV VP1 VLPs as determined by an indirect ELISA and Western blot.

Clone	Isotype	MAb Reactivity with:					
		TSPyV VP1 (Western Blot)	TSPyV VP1 (ELISA)	TSVP1-PS1-1 (ELISA)	TSVP1-PADRE-1 (ELISA)	TSVP1-PS1-4 (ELISA)	TSVP1-PADRE-4 (ELISA)
**2B4**	IgG2a	-	+	-	-	+	+
**7A2**	IgG1	-	+	+	+	+	+
**9F2**	IgG1	-	+	+	+	+	+
**9D3**	IgG1	-	+	+	+	+	+
**17G11**	IgG2a	-	+	+	+	+	+
**18A7**	IgG2a	-	+	+	+	-	-
**19E4**	IgG1	-	+	+	+	+	+
**20D3**	IgG1	-	+	+	+	+	+
**5E6**	IgG2a	-	+	-	-	-	-
**16H6**	IgG2a	-	+	+	+	+	+
**3C5**	IgG1	+	+	+	+	+	+
**4A1**	IgG1	+	+	+	+	+	+
**8C4**	IgG1	+	+	+	+	+	+
**11F12**	IgG1	+	+	+	+	+	+
**16A5**	IgG1	+	+	+	+	+	+
**17A5**	IgG1	+	+	+	+	+	+
**19A5**	IgG2a	+	+	+	+	+	+

Western blot reactivity: (+) positive, stained protein band of expected molecular weight; (−) negative, no protein band stained; The reactivity in ELISA: (+) positive, OD > 1.0; (-) negative, OD < 0.1; For the ELISA test, optimal dilution (1:20) of hybridoma supernatants was used at which the highest specific immunostaining and no background staining was observed.

Table S3. Titers of VLP-specific (rows 1–10) and insert-specific (rows 11–12) IgG antibodies determined by an indirect ELISA after a primary immunization in groups of BALB/c mice (n = 3) immunized with different chimeric VLPs emulsified in a complete Freund adjuvant.

No.	Antigen in ELISA:	Antigen Used for Immunization:							
		TSVP1-PS1-1	TSVP1-PS1-4	TSVP1-PADRE-1	TSVP1-PADRE-4	HaVP1-PS1-1	HaVP1-PS1-4	HaVP1-PADRE-1	HaVP1-PADRE-4
**1.**	**TSVP1- PS1-1**	108,200	82,600						
**2.**	**TSVP1-PS1-4**	87,500	104,600						
**3.**	**TSVP1-PADRE-1**			31,100	3600				
**4.**	**TSVP1-PADRE-4**			4900	950				
**5.**	**HaVP1-PS1-1**					47,257	7100		
**6.**	**HAVP1-PS1-4**					14,900	16,400		
**7.**	**HaVP1-PADRE-1**							98,400	104,200
**8.**	**HaVP1-PADRE-4**							7500	31,200
**9.**	**TSPyV VP1**	21,500	30,900	14,300	900				
**10.**	**HaPyV VP1**					4100	3600	29,000	15,600
**11.**	**PS1 peptide**	2600	3900			1200	400		
**12.**	**PADRE peptide**			1300	300			4700	14,000

Antibody titer is defined as the reciprocal of the highest antiserum dilution giving OD_{450} value greater than three times the background that corresponds to the mean OD_{450} + 3SD of a the preimmune serum diluted 1:100.

© 2015 by the authors; licensee MDPI, Basel, Switzerland. This article is an open access article distributed under the terms and conditions of the Creative Commons Attribution license (http://creativecommons.org/licenses/by/4.0/).
